# Effects of Airborne Particulate Matter on Respiratory Morbidity in Asthmatic Children

**DOI:** 10.2188/jea.JE2007432

**Published:** 2008-05-29

**Authors:** Lu Ma, Masayuki Shima, Yoshiko Yoda, Hirono Yamamoto, Satoshi Nakai, Kenji Tamura, Hiroshi Nitta, Hiroko Watanabe, Toshiyuki Nishimuta

**Affiliations:** 1 Department of Public Health, Hyogo College of Medicine.; 2 Graduate School of Environment and Information Sciences, Yokohama National University.; 3 Environmental Health Science Division, National Institute for Environmental Studies.; 4 Department of Pediatrics, Shimoshizu National Hospital.

**Keywords:** Particulate Matter, Asthma, Peak Expiratory Flow Rate, Respiratory Sounds

## Abstract

**Background:**

The effects of airborne particulate matter (PM) are a major human health concern. In this panel study, we evaluated the acute effects of exposure to PM on peak expiratory flow (PEF) and wheezing in children.

**Methods:**

Daily PEF and wheezing were examined in 19 asthmatic children who were hospitalized in a suburban city in Japan for approximately 5 months. The concentrations of PM less than 2.5 µm in diameter (PM_2.5_) were monitored at a monitoring station proximal to the hospital. Moreover, PM_2.5_ concentrations inside and outside the hospital were measured using the dust monitor with a laser diode (PM_2.5(LD)_). The changes in PEF and wheezing associated with PM concentration were analyzed.

**Results:**

The changes in PEF in the morning and evening were significantly associated with increases in the average concentration of indoor PM_2.5(LD)_ 24 h prior to measurement (-2.86 L/min [95%CI: -4.12, -1.61] and -3.59 L/min [95%CI: -4.99, -2.20] respectively, for 10-µg/m^3^ increases). The change in PEF was also significantly associated with outdoor PM_2.5(LD)_ concentrations, but the changes were smaller than those observed for indoor PM_2.5(LD)_. Changes in PEF and concentration of stationary-site PM_2.5_ were not associated. The prevalence of wheezing in the morning and evening were also significantly associated with indoor PM_2.5(LD)_ concentrations (odds ratios = 1.014 [95%CI: 1.006, 1.023] and 1.025 [95%CI: 1.013, 1.038] respectively, for 10-µg/m^3^ increases). Wheezing in the evening was significantly associated with outdoor PM_2.5(LD)_ concentration. The effects of indoor and outdoor PM_2.5(LD)_ remained significant even after adjusting for ambient nitrogen dioxide concentrations.

**Conclusion:**

Indoor and outdoor PM_2.5(LD)_ concentrations were associated with PEF and wheezing among asthmatic children. Indoor PM_2.5(LD)_ had a more marked effect than outdoor PM_2.5(LD)_ or stationary-site PM_2.5_.

## INTRODUCTION

The effects of airborne particulate matter (PM) on human health have become a major concern.^1^^-^^3^ Numerous previous panel studies have evaluated the acute effects of short-term exposure to PM on exacerbation of asthma in children in Western countries.^4^^-^^14^ These studies have reported a relationship between elevated concentrations of PM and an increase in respiratory symptoms^4^^-^^10^ as well as decreased pulmonary function values.^5^^,^^8^^,^^9^^,^^11^^-^^14^ Many of these studies have examined the effects of PM with aerodynamic diameter less than 10 µm (PM_10_). Recently, it has been reported that fine particles may have more adverse effects on respiratory symptoms and pulmonary functions than coarse particles.^15^^,^^16^ Air quality standards for atmospheric concentrations of PM less than 2.5 µm in diameter (PM_2.5_) have been established in the United States and European countries.^17^ In Japan, many epidemiologic researches have dealt with the chronic effects of long-term exposure to air pollutants.^18^^-^^20^ However, only a few studies have investigated the acute effects of short-term exposure to PM.^21^^,^^22^

With respect to exposure assessment, in most previous studies, subjects were usually assigned concentrations measured at central regional sites or other outdoor sites. Use of central regional PM concentrations may lead to exposure misclassification and diminish the accuracy of exposure-response estimates. Many people spend most of their time indoors, where they are exposed to a combination of indoor-generated PM and outdoor-originated PM that has infiltrated the house.^23^^,^^24^ Indoor concentrations of PM often differ from outdoor PM concentrations.^24^^-^^26^ Therefore, to improve the accuracy of the estimated associations, concentrations of PM in the environment in which the subjects spend the majority of their time should be evaluated.

In this panel study, we evaluated the potential relationship between exposure to PM and asthma exacerbation in children who were hospitalized in a suburban city in Japan. The concentrations of PM were monitored inside and outside the hospital and at a monitoring station proximal to the hospital. To assess the acute effects of PM, we evaluated peak expiratory flow (PEF) and wheezing in the children.

## METHODS

### Subjects

The subjects of this panel study were 19 children aged 8-15 years, who had physician-diagnosed severe asthma and were hospitalized at Shimoshizu National Hospital in Yotsukaido City, Chiba Prefecture, Japan. Because the children had poorly controlled asthma with frequent exacerbations, they were under long-term hospitalization for maintenance medication for asthma, and attended a school for sick children, which was adjacent to the hospital. In November 2003, 19 children were under long-term hospitalization, and informed written consent was obtained from all the subjects and their parents. All of them had atopic disposition and received asthma medication, including inhaled corticosteroids (ICS). No major roads or factories were present in the vicinity of the hospital. This study was approved by the Medical Ethics Committee of Shimoshizu National Hospital.

### Health Outcomes

PEF of all the children was evaluated daily using an electronic spirometer (AS-300; Minato Medical Science Inc., Tokyo, Japan). The measurements were conducted immediately prior to medication at least twice a day, i.e., in the morning (6:00 AM) and evening (7:00 PM), under the guidance of trained nurses. The presence or absence of wheezing was assessed based on auscultation by the trained nurses, and recorded with the results of PEF. For this study, we collected the records from November 5, 2003 through March 24, 2004.

### Particulate Matter Measurements

To measure PM concentrations inside and outside the hospital, we used a digital dust monitor (LD-3K; Sibata Scientific Technology Inc., Tokyo, Japan), which is a portable monitor based on the light scattering principle, with a laser diode as the light source. The monitor determines the relative concentrations of PM by measuring the intensity of the laser beam scattered by particles. To convert the relative concentrations to mass concentrations of PM, conversion coefficients must be calculated based on the mass concentrations measured simultaneously using the filtration sampling method. We measured the mass concentrations of PM 7 times over a period of 24 h by using collocated portable air samplers (MP-Σ300; Sibata Scientific Technology Inc., Tokyo, Japan) equipped with cascade impactors (ATPS-20H; Sibata Scientific Technology Inc., Tokyo, Japan) with a flow rate of 1.5 L/min; the cut-off points of aerodynamic diameter were 2.5 µm and 10 µm (PM_2.5_ and PM_2.5-10_ respectively). The measurements by LD-3K strongly correlated with the concentrations of PM_2.5_, and the R^2^ value between them was 0.99 in the ward of the hospital and 0.93 at the entrance of the hospital, when compared with the mass concentrations based on the filtration sampling method. However, the measurements by LD-3K were not highly associated with the concentrations of coarse particles (PM_2.5-10_) (R^2^ = 0.62 and 0.51 in the ward of the hospital and at the entrance, respectively). Therefore, the respective conversion coefficients were calculated for PM concentrations inside or outside the hospital based on the relationship of the measurements of LD-3K to the mass concentrations of PM_2.5_. The values (PM_2.5(LD)_) converted by the coefficients were considered to be the approximate PM_2.5_ concentrations.

During the study period, PM concentrations were continuously monitored using LD-3K in 2 hospital rooms, a hall in the children’s ward, and at the entrance of the hospital. The average concentration of PM at the 3 sites in the hospital (2 hospital rooms and the hall of the ward) was regarded as the indoor PM_2.5(LD)_ concentration, while the concentration at the entrance was regarded as the outdoor PM_2.5(LD)_ concentration.

In addition, the concentration of PM_2.5_ was measured with a tapered-element oscillating microbalance (TEOM; Thermo Electron Inc., East Greenbush, NY, USA) at a monitoring station proximal to the hospital. The concentrations of nitrogen dioxide (NO_2_), temperature, and relative humidity were also measured continuously at the station. The distance between the hospital and the station was approximately 500 m.

### Data Analysis

We used descriptive statistics of PM and NO_2_ concentrations, temperature, and relative humidity, evaluated by correlation matrices for them. We examined daily measurements of PEF and wheezing in the asthmatic children in relation to the concentrations of PM and NO_2_.

For regression analyses of daily PEF, we used the Generalized Estimating Equation (GEE),^27^ which is suitable for correlated data in individuals.^28^ The standard error of the regression estimate is adjusted for the possible correlation among the responses from 1 subject. This method generates robust estimators regardless of the specifications of the covariance matrix, and as autocorrelation is included in the covariance, coefficients can be interpreted as usual. The analyses for the measurements in the morning and evening were performed separately using each model. The results are demonstrated as the mean changes in PEF for 10-µg/m^3^ or 10-ppb increments of PM or NO_2_, respectively, after adjustment for sex, age (months), height (in November 2003), temperature, relative humidity, and growth of the children. Because only the heights of the children at the beginning of this study were available, we applied an ordinal variable, i.e., 1-5, to each month during this study (November 2003 through March 2004) as a surrogate for their growth. The minimum and maximum temperatures during the day were included in the model for the analyses of PEF in the morning and evening, respectively.

Exposure variables included the average concentration of each pollutant during the 12- or 24-h period preceding measurement. We also evaluated the effect of the 1-h maximum concentration of each pollutant in the 12-h period preceding measurement. We first performed the analyses with a single-pollutant model. Second, we carried out the analyses using 2-pollutant models, including NO_2_ and one of the PM concentrations. Thereafter, to assess the potential of the delayed effects of PM, we also examined the effects of PM concentration on certain days before the day of PEF measurement; the number of days preceding measurement was termed as the number of lag days. This was accomplished by regressing PEF on PM concentrations measured every 24 or 12 h, upto 3 lag days.

We also used the GEE for analyzing the effects of pollutants on wheezing. Effect estimates for wheezing were expressed as odds ratios (ORs) with 95% confidence intervals (CIs) for 10-µg/m^3^ or 10-ppb increments of PM or NO_2_, respectively, after adjustment for sex, age (months), temperature, and relative humidity. In addition, the concentrations of each PM were categorized into quartiles and included in the model as dummy variables. The ORs and 95% CIs were calculated relative to the lowest quartile of each PM. The other procedures used for the analyses of wheezing were similar to those used for the analyses of PEF.

All statistical analyses were performed using the SPSS^®^ 15.0 software (SPSS Inc., Chicago, IL, USA).

## RESULTS

### Descriptive Statistics

**Figure 1.**
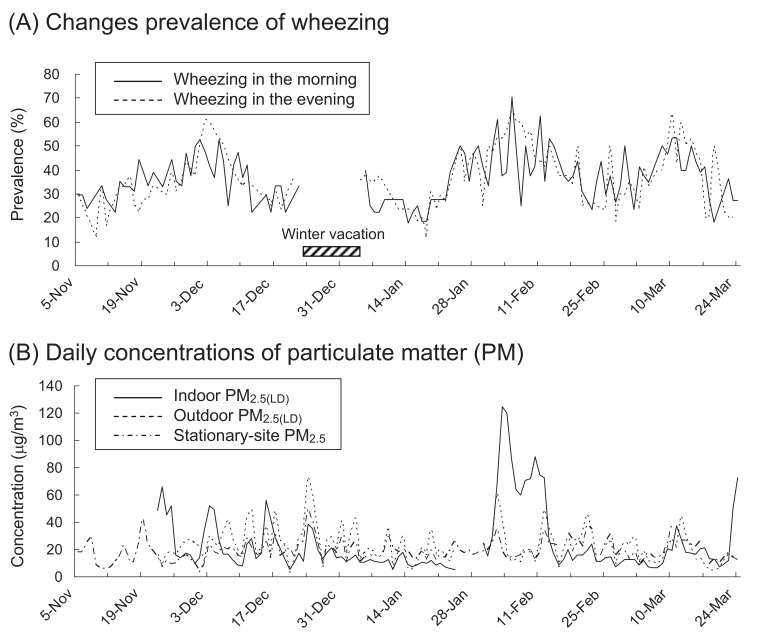
Daily prevalence of wheezing in the morning and evening (A) and daily concentrations of indoor and outdoor PM_2.5(LD)_ and stationary-site PM_2.5_ (24-h means) (B), from November 5, 2003 through March 24, 2004. PM_2.5_, particulate matter less than 2.5 µm in diameter; PM_2.5(LD)_, PM_2.5_ measured using the dust monitor with a laser diode.

Table 1 shows the characteristics of the subjects and summarizes the daily measurements of PEF and records of wheezing in this study. The study population comprised 19 children (8 boys and 11 girls). The mean (standard deviation) PEF was 288.9 (75.5) L/min in the morning and 306.5 (75.1) L/min in the evening. The prevalence of wheezing, as noted in the medical records of the children, was 35.7% in the morning and 35.0% in the evening. Daily prevalence of wheezing is shown in Figure 1. In February 2004, a somewhat higher prevalence of wheezing was observed.

**Table 1.** Descriptive statistics for the study subjects on the basis of sex.

	Male	Female	Total
	(n = 8)	(n = 11)	(n = 19)
Mean age (SD)(years)	12.4 (2.2)	13.3 (2.5)	12.9 (2.4)

Mean height^*^ (SD) (cm)	150.6 (19.3)	147.4 (11.9)	148.8 (15.1)

Number of PEF measurements			
Morning, mean (SD)	81.9 (22.9)	88.4 (8.4)	85.6 (16.0)
Evening, mean (SD)	82.8 (22.0)	90.6 (6.9)	87.1 (15.5)

PEF			
Morning PEF, mean (SD) (L/min)	322.6 (88.2)	264.4 (57.1)	288.9 (75.5)
Evening PEF, mean (SD) (L/min)	333.7 (96.9)	284.8 (46.6)	306.5 (75.1)

Prevalence of wheezing			
Percentage in the morning (%)	33.8	37.2	35.7
Percentage in the evening (%)	36.4	33.8	35.0

SD, Standard deviation; PEF, peak expiratory flow.

* : Height in November 2003.

Table 2 describes the concentrations of PM and NO_2_. The mean concentration of indoor PM_2.5(LD)_ was higher during the nighttime than during the daytime. In comparison, the mean concentrations of outdoor PM_2.5(LD)_ and stationary-site PM_2.5_ during the daytime were similar to those during the nighttime. Daily concentrations of indoor and outdoor PM_2.5(LD)_ and stationary-site PM_2.5_ are also shown in Figure 1. At the beginning of February 2004, the concentrations of indoor PM_2.5(LD)_ were considerably high. The concentration of outdoor PM_2.5(LD)_ was moderately correlated with the concentrations of stationary-site PM_2.5_ and NO_2_ (Table 3). However, the concentration of indoor PM_2.5(LD)_ showed weak correlation with outdoor PM_2.5(LD)_ concentration and no correlation with the concentrations of stationary-site PM_2.5_ and NO_2_.

**Table 2.** Descriptive statistics of daily measurements of air pollutants and temperature during the study period.

Pollutants/temperature	n^*^	Mean	SD	Minimum	Median	Maximum
Indoor PM_2.5(LD)_ (µg/m^3^)						
24-h mean	119	24.6	23.2	5.3	15.3	124.5
12-h nighttime mean	119	28.3	33.8	4.5	15.2	181.1
12-h daytime mean	120	21.7	17.1	3.5	15.9	103.1
1-h nighttime maximum	119	44.7	52.1	5.5	23.8	238.9
1-h daytime maximum	120	41.5	42.2	4.6	24.3	192.9

Outdoor PM_2.5(LD)_ (µg/m^3^)						
24-h mean	118	22.6	12.7	3.3	20.3	73.5
12-h nighttime mean	118	21.7	16.3	1.7	16.7	92.1
12-h daytime mean	120	23.6	14.1	4.3	20.9	78.5
1-h nighttime maximum	118	34.0	23.6	2.9	29.0	136.4
1-h daytime maximum	120	38.6	22.6	5.6	35.0	110.9

Stationary-site PM_2.5_ (TEOM)(µg/m^3^)						
24-h mean	136	19.1	7.8	3.5	18.1	49.9
12-h nighttime mean	141	17.2	8.5	2.6	16.7	56.4
12-h daytime mean	136	21.2	10.4	2.9	18.3	56.2
1-h nighttime maximum	141	28.5	14.8	4.9	25.6	95.8
1-h daytime maximum	136	35.8	15.8	4.9	33.7	110.8

Stationary-site NO_2_ (ppb)						
24-h mean	141	20.6	7.7	6.5	21.1	41.0
12-h nighttime mean	141	21.8	9.7	3.3	21.1	42.6
12-h daytime mean	141	19.3	8.9	4.3	18.2	41.2
1-h nighttime maximum	141	31.6	11.9	5.0	32.0	57.0
1-h daytime maximum	141	31.5	11.9	5.0	32.0	56.0

Stationary-site temperature (°C)						
1-h maximum temperature	141	12.9	3.8	4.6	12.4	23.9
1-h minimum temperature	141	5.1	3.7	-0.8	4.2	15.2

Stationary-site relative humidity (%)						
24-h mean	141	61.4	15.9	28.0	60.0	93.0

SD, Standard deviation; PM_2.5_, particulate matter less than 2.5 µm in diameter; PM_2.5(LD)_, PM_2.5_ measured using the dust monitor with a laser diode; TEOM, tapered-element oscillating microbalance; NO_2_, nitrogen dioxide.

* : Some data are missing due to inefficient maintenance of the monitors or power failure.

**Table 3.** Correlation matrix of daily pollutants and temperature during the study period.

	Indoor PM_2.5(LD)_, 24-h mean	Outdoor PM_2.5(LD)_, 24-h mean	Stationary-site PM_2.5_, 24-h mean	Stationary-site NO_2_, 24-h mean	1-h maximum temperature	1-h minimum temperature	Relative humidity, 24-h mean
Indoor PM_2.5(LD)_, 24-h mean	1	0.187 ^*^	0.031	0.137	0.008	0.076	0.193 ^*^
Outdoor PM_2.5(LD)_, 24-h mean		1	0.674 ^**^	0.585 ^**^	0.155	0.132	0.393 ^**^
Stationary-site PM_2.5_, 24-h mean			1	0.473 ^**^	0.173 ^*^	-0.040	-0.051
Stationary-site NO_2_, 24-h mean				1	-0.018	-0.123	0.053
1-h maximum temperature					1	0.775 ^**^	0.300 ^**^
1-h minimum temperature						1	0.527 ^**^
Relative humidity, 24-h mean							1

The number of observations is 141 for stationary-site NO_2_, temperature, and relative humidity; 136 for stationary-site PM_2.5_; 119 for indoor PM_2.5(LD)_; and 118 for outdoor PM_2.5(LD)_.

PM_2.5_, particulate matter less than 2.5 µm in diameter; PM_2.5(LD)_, PM_2.5_ measured using the dust monitor with a laser diode; NO_2_, nitrogen dioxide.

* : *P*<0.05, ** : *P*<0.01

### Peak Expiratory Flow and Exposure to Particulate Matter

Table 4 shows the changes in PEF associated with a 10-µg/m^3^ or 10-ppb increment in the concentration of each pollutant, using single-pollutant models adjusted for sex, age, height, temperature, relative humidity, and growth of the children. In 2-pollutant models including NO_2_ concentration, in addition to the above factors, the changes in PEF in the morning and evening were also significantly associated with the increase in average concentrations of indoor PM_2.5(LD)_ during the 24-h lag period. The changes in PEF were also significantly associated with the average concentration and 1-h maximum concentration of indoor PM_2.5(LD)_ in the preceding 12 h. The change in PEF in the evening was larger than that in the morning. Moreover, some significant associations were present between the change in PEF and outdoor PM_2.5(LD)_ concentrations, but the changes were smaller in relation to indoor PM_2.5(LD)_ concentrations. The changes in PEF were not associated with stationary-site PM_2.5_ or NO_2_ concentrations. Two-pollutant models adjusted for NO_2_ concentration showed similar associations between changes in PEF and PM concentrations.

**Table 4.** Estimates and 95% confidence intervals (CIs) for change in peak expiratory flow (PEF) per 10-µg/m^3^ or 10-ppb increase of each pollutant during the study period.

	Single-pollutant model^*^				Two-pollutant model^†^			
	
	Change ^‡^	95% CI		*P* value	Change ^‡^	95% CI		*P* value
	PEF in the morning							
Indoor PM_2.5(LD)_								
24-h mean	-2.86	-4.12	-1.61	<0.001	-2.92	-4.23	-1.61	<0.001
12-h mean	-2.11	-3.02	-1.21	<0.001	-2.12	-3.04	-1.20	<0.001
1-h maximum in the preceding 12 h	-1.42	-2.03	-0.82	<0.001	-1.42	-2.03	-0.82	<0.001

Outdoor PM_2.5(LD)_								
24-h mean	-1.34	-2.99	0.32	0.113	-1.96	-3.84	-0.09	0.040
12-h mean	-1.65	-3.18	-0.12	0.034	-2.04	-3.64	-0.44	0.013
1-h maximum in the preceding 12 h	-1.51	-2.59	-0.43	0.006	-1.88	-3.06	-0.69	0.002

Stationary-site PM_2.5_								
24-h mean	-0.35	-1.89	1.20	0.662	0.01	-1.61	1.63	0.991
12-h mean	-0.54	-2.99	1.92	0.667	-0.55	-3.20	2.10	0.685
1-h maximum in the preceding 12 h	-1.03	-2.24	0.19	0.098	-1.34	-2.80	0.13	0.074

Stationary-site NO_2_								
24-h mean	-0.68	-2.65	1.29	0.498	-			
12-h mean	-0.26	-1.96	1.44	0.761	-			
1-h maximum in the preceding 12 h	0.03	-1.21	1.26	0.968	-			

	PEF in the evening							
Indoor PM_2.5(LD)_								
24-h mean	-3.59	-4.99	-2.20	<0.001	-3.59	-4.98	-2.20	<0.001
12-h mean	-4.92	-7.00	-2.85	<0.001	-4.96	-7.04	-2.89	<0.001
1-h maximum in the preceding 12 h	-2.22	-3.09	-1.36	<0.001	-2.23	-3.10	-1.37	<0.001

Outdoor PM_2.5(LD)_								
24-h mean	-3.40	-6.47	-0.33	0.030	-4.00	-7.51	-0.49	0.025
12-h mean	-1.87	-3.85	0.11	0.064	-2.39	-4.75	-0.02	0.048
1-h maximum in the preceding 12 h	-0.65	-1.69	0.38	0.217	-0.48	-1.35	0.39	0.283

Stationary-site PM_2.5_								
24-h mean	-1.38	-3.84	1.08	0.271	-0.28	-2.63	2.06	0.812
12-h mean	-0.72	-2.43	0.98	0.406	-0.80	-2.60	1.01	0.388
1-h maximum in the preceding 12 h	-0.73	-1.85	0.39	0.202	-0.45	-1.48	0.58	0.393

Stationary-site NO_2_								
24-h mean	-1.69	-4.18	0.81	0.186	-			
12-h mean	-0.34	-2.66	1.98	0.774	-			
1-h maximum in the preceding 12 h	-1.27	-2.91	0.38	0.131	-			

PM_2.5_, particulate matter less than 2.5 µm in diameter; PM_2.5(LD)_, PM_2.5_ measured using the dust monitor with a laser diode; NO_2_, nitrogen dioxide.

* : The association between PEF and each pollutant was analyzed and adjusted for sex, age, height, temperature, relative humidity, and growth of the children.

† : The association between PEF and each pollutant was analyzed and adjusted for NO_2_ concentration, sex, age, height, temperature, relative humidity, and growth of the children.

‡ : Mean changes in PEF (L/min) associated with a 10 µg/m^3^ or 10 ppb increase of each pollutant.

**Figure 2.**
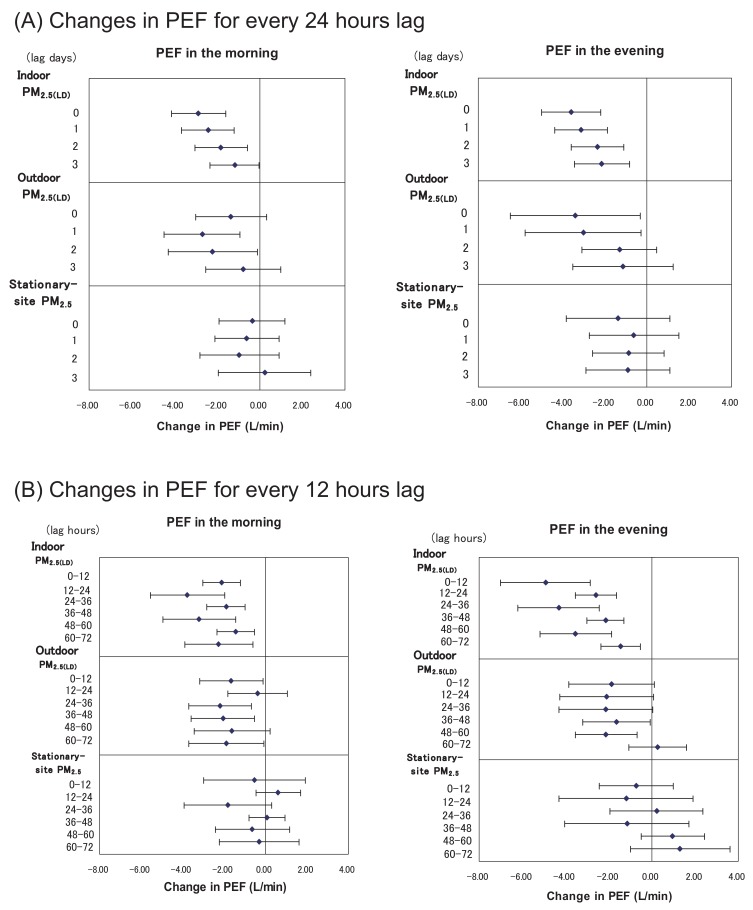
Changes in peak expiratory flow (PEF) in relation to the concentration of particulate matter (PM) for every 24 hours (A) and 12 hours (B), up to 3 days before the measurement (3 lag days). Estimates for changes in PEF with 95% confidence intervals are shown per 10 µg/m^3^ increase of each PM, adjusted for sex, age, height, temperature, relative humidity, and growth of the children.

Figure 2 shows the changes in PEF in relation to the average concentrations of PM for every 24 h, upto 3 lag days. The largest decreases in PEF in relation to the concentrations of indoor PM_2.5(LD)_ were recorded for the morning and evening of the same day. The effects of indoor PM_2.5(LD)_ on PEF were significant for upto 3 d in the morning and evening; however, the decreases in PEF became gradually smaller as the number of lag days increased. However, for a 1-d lag, the decrease in PEF in relation to the concentration of outdoor PM_2.5(LD)_ was larger than that for the day of measurement. The associations were significant for 1- and 2-d lags in the morning and 0- and 1-d lags in the evening. No significant effects of stationary-site PM_2.5_ on PEF were observed on the same day or upto 3 lag days either the morning or in the evening.

The changes in PEF in relation to the average concentrations of PM for every 12 h prior to measurement are also shown in Figure 2. Consistent decreases in PEF in the morning and evening were observed in relation to increases in indoor PM_2.5(LD)_ concentrations, upto 72 h prior to measurement. PEF showed the greatest decrease in the morning in relation to the indoor PM_2.5(LD)_ concentration during the 12-24-h lag period. However, PEF showed the greatest decrease in the evening in relation to the indoor PM_2.5(LD)_ concentration during the preceding 0-12 h. Thus, the changes in PEF in relation to daytime indoor PM_2.5(LD)_ concentrations were greater than those in relation to nighttime indoor PM_2.5(LD)_ concentrations. With regard to the effect of outdoor PM_2.5(LD)_ on PEF, the decreases in PEF in the morning and evening were greatest during the 24-36-h lag period. The changes in PEF were not related to the concentrations of stationary-site PM_2.5_ during the 0-72-h lag period.

### Wheezing and Exposure to Particulate Matter

Table 5 shows ORs for wheezing associated with a 10-µg/m^3^ or 10-ppb increment of each pollutant. The prevalence of wheezing in the morning and evening was significantly associated with the average concentration of indoor PM_2.5(LD)_ in the 24-h lag period. Wheezing was also significantly associated with both the average and 1-h maximum concentrations of indoor PM_2.5(LD)_ in the preceding 12 h. The associations were stronger in the evening than in the morning. Wheezing in the evening was significantly associated with the average concentrations of outdoor PM_2.5(LD)_ in the preceding 24 or 12 h. A few significant associations were also present between wheezing in the evening and stationary-site PM_2.5_. However, the associations between wheezing and stationary-site PM_2.5_ were not significant in the 2-pollutant models adjusted for NO_2_ concentration. Table 6 shows the ORs for wheezing relative to the lowest quartile of each PM in the preceding 24 h. Indoor PM_2.5(LD)_ concentrations of 15.4 µg/m^3^ or higher were significantly associated with increased wheezing in the morning. Wheezing in the evening was associated with indoor PM_2.5(LD)_ concentrations ≥11.0 µg/m^3^ and stationary-site PM_2.5_ concentrations ≥18.2 µg/m^3^.

**Table 5.** Adjusted odds ratios (ORs) and 95% confidence intervals (CIs) for wheezing per 10-µg/m^3^ or 10-ppb increase in each pollutant during the study period.

	Single-pollutant model^*^				Two-pollutant model ^†^			
	
	OR ^‡^	95% CI		*P* value	OR ^‡^	95% CI		*P* value
	Wheezing in the morning							
Indoor PM_2.5(LD)_								
24-h mean	1.014	1.006	1.023	<0.001	1.015	1.006	1.024	<0.001
12-h mean	1.011	1.005	1.016	<0.001	1.011	1.005	1.017	<0.001
1-h maximum in the preceding 12 h	1.007	1.004	1.011	<0.001	1.007	1.004	1.011	<0.001

Outdoor PM_2.5(LD)_								
24-h mean	0.993	0.980	1.006	0.271	0.997	0.983	1.010	0.624
12-h mean	1.001	0.988	1.014	0.888	1.002	0.990	1.015	0.707
1-h maximum in the preceding 12 h	1.003	0.994	1.011	0.559	1.003	0.993	1.012	0.591

Stationary-site PM_2.5_								
24-h mean	1.014	0.987	1.042	0.301	1.020	0.978	1.063	0.363
12-h mean	1.013	1.000	1.026	0.052	1.020	0.995	1.046	0.119
1-h maximum in the preceding 12 h	1.014	0.997	1.031	0.119	1.015	0.993	1.038	0.184

Stationary-site NO_2_								
24-h mean	0.995	0.971	1.019	0.670	-			
12-h mean	0.998	0.979	1.016	0.808	-			
1-h maximum in the preceding 12 h	1.002	0.991	1.014	0.675	-			

	Wheezing in the evening							
Indoor PM_2.5(LD)_								
24-h mean	1.025	1.013	1.038	<0.001	1.025	1.012	1.038	<0.001
12-h mean	1.040	1.020	1.060	<0.001	1.040	1.020	1.062	<0.001
1-h maximum in the preceding 12 h	1.016	1.008	1.024	<0.001	1.016	1.008	1.025	<0.001

Outdoor PM_2.5(LD)_								
24-h mean	1.016	1.002	1.029	0.024	1.010	0.996	1.026	0.170
12-h mean	1.014	1.002	1.026	0.022	1.017	1.001	1.033	0.041
1-h maximum in the preceding 12 h	1.002	0.992	1.011	0.739	0.998	0.988	1.009	0.764

Stationary-site PM_2.5_								
24-h mean	1.033	1.008	1.058	0.009	1.027	0.984	1.073	0.219
12-h mean	1.022	1.004	1.042	0.019	1.024	0.994	1.055	0.116
1-h maximum in the preceding 12 h	1.006	0.997	1.016	0.177	1.002	0.990	1.014	0.700

Stationary-site NO_2_								
24-h mean	1.024	0.996	1.052	0.093	-			
12-h mean	1.011	0.990	1.033	0.293	-			
1-h maximum in the preceding 12 h	1.014	1.001	1.028	0.035	-			

PM_2.5_, particulate matter less than 2.5 µm in diameter; PM_2.5(LD)_, PM_2.5_ measured using the dust monitor with a laser diode; NO_2_, nitrogen dioxide.

* : The association between wheezing and each pollutant was analyzed and adjusted for sex, age, temperature, and relative humidity.

† : The association between wheezing and each pollutant was analyzed and adjusted for NO_2_ concentration, sex, age, temperature, and relative humidity.

‡ : ORs for wheezing associated with a 10 µg/m^3^ or 10 ppb increase of each pollutant.

**Table 6.** Adjusted odds ratios (ORs) and 95% confidence intervals (CIs) for wheezing, in relation to quatiles of 24-h mean concentrations of each particulate matter (PM) during the study period.

	Wheezing in the morning			Wheezing in the evening		
	
	OR^*^	95% CI		OR^*^	95% CI	
Indoor PM_2.5(LD)_, 24-h mean (µg/m^3^)						
<11.0	1.000			1.000		
11.0-15.3	1.053	0.989	1.121	1.098	1.038	1.161
15.4-27.9	1.092	1.034	1.153	1.137	1.052	1.228
≥28.0	1.081	1.021	1.144	1.217	1.100	1.345
*P* value	0.011			0.002		

Outdoor PM_2.5(LD)_, 24-h mean (µg/m^3^)						
<13.0	1.000			1.000		
13.0-20.3	0.960	0.895	1.029	1.022	0.954	1.094
20.4-28.9	0.954	0.910	1.001	1.022	0.983	1.063
≥29.0	0.983	0.940	1.029	1.035	0.990	1.081
*P* value	0.208			0.474		

Stationary-site PM_2.5_, 24-h mean (µg/m^3^)						
<13.9	1.000			1.000		
13.9-18.1	1.029	0.960	1.103	1.010	0.957	1.067
18.2-23.5	1.015	0.957	1.077	1.062	1.017	1.109
≥23.6	1.015	0.947	1.088	1.094	1.032	1.160
*P* value	0.822			0.010		

PM_2.5_, particulate matter less than 2.5 µm in diameter; PM_2.5(LD)_, PM_2.5_ measured using the dust monitor with a laser diode.

* : ORs for wheezing relative to the lowest quartile of each PM, adjusted for sex, age, temperature, and relative humidity.

**Figure 3.**
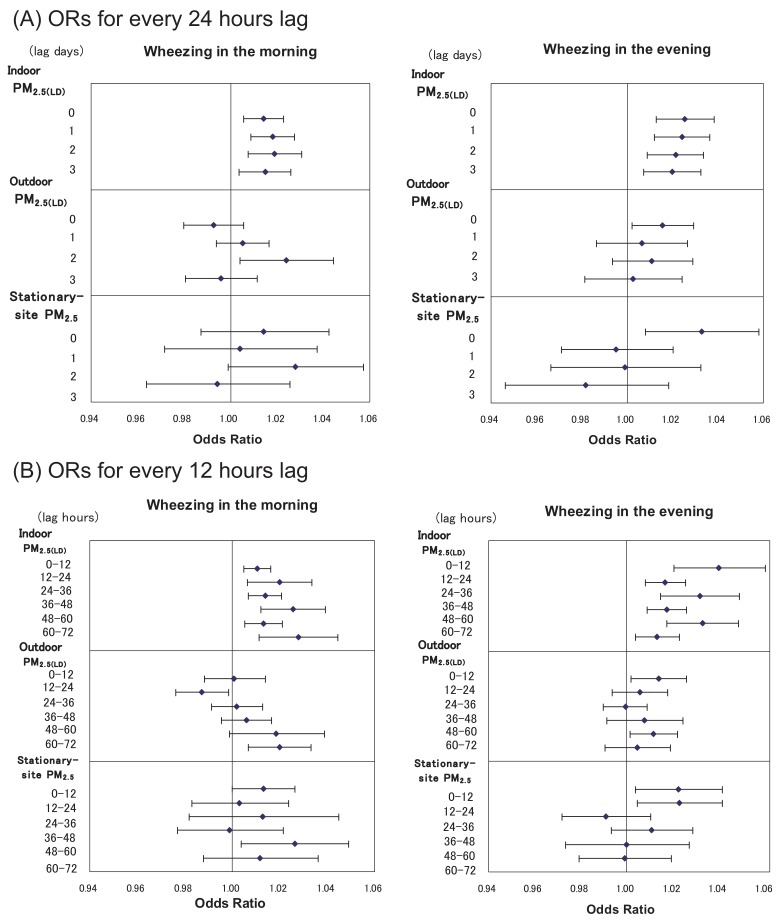
Odds ratios (ORs) for wheezing in relation to the concentration of particulate matter (PM) for every 24 hours (A) and 12 hours (B), up to 3 days before the measurement (3 lag days). ORs with 95% confidence intervals are shown per 10 µg/m^3^ increase of each PM, adjusted for sex, age, temperature, and relative humidity.

Figure 3 shows the associations between wheezing and the average concentrations of PM for every 24 h upto 3 lag days. The prevalence of wheezing in the morning and evening significantly increased in relation to the increase in indoor PM_2.5(LD)_ concentrations for 0-3 d lags. The association of ORs for wheezing in the morning with outdoor PM_2.5(LD)_ gradually increased for 0-2 d lags, and the association with the 2-d lag was significant. The association between wheezing in the evening and outdoor PM_2.5(LD)_ was significant only for the same day. Wheezing in the evening was also significantly related to the concentration of stationary-site PM_2.5_ on the same day.

The association of wheezing with the average concentrations of PM for every 12 h preceding measurement is also shown in Figure 3. In the morning and evening, the associations between wheezing and indoor PM_2.5(LD)_ concentrations were consistently significant for upto 72 h prior to measurement. The effects of indoor PM_2.5(LD)_ concentration on wheezing were greater during the daytime than during nighttime. Some significant associations were present between wheezing and the concentrations of outdoor PM_2.5(LD)_ and stationary-site PM_2.5_, although these associations were not consistently observed.

## DISCUSSION

In this panel study, we evaluated the acute effects of short-term exposure to PM by daily measurements of PEF and wheezing among asthmatic children in a hospital in a suburban city. All the children had been hospitalized for several months and attended a school for sick children, which was adjacent to the hospital. Because they spent almost the entire day in the hospital or school, their exposure levels to PM were considered to be nearly equal. Although all the children received asthma medication including ICS, they showed significant decreases in PEF and increases in wheezing after indoor or outdoor PM_2.5(LD)_ concentrations were elevated. In particular, PEF and wheezing were shown to have consistent and strong associations with indoor PM_2.5(LD)_ concentrations. The effects of indoor and outdoor PM_2.5(LD)_ concentrations remained significant even after adjusting for ambient NO^2^ concentrations.

Numerous studies have previously reported that PEF among asthmatics significantly decreases in relation to an increase in daily PM concentration.^5^^,^^8^^,^^9^^,^^11^^,^^13^^,^^15^^,^^29^ With respect to the effects of fine particles, Romieu et al^9^ evaluated the changes in daily PEF in relation to increases in PM_2.5_ in a panel of asthmatic children (aged 5-7 years), and observed significant decreases in PEF in the morning and evening (-2.36 L/min [95% CI: -3.86, -0.86] and -1.71 L/min [95% CI: -3.09, -0.34] respectively for a 10-µg/m^3^ increase in the 24-h concentrations of PM_2.5_). Many studies have also evaluated respiratory symptoms in asthmatics in relation to exposure to PM.^4^^-^^10^^,^^29^^,^^30^ Romieu et al^9^ reported that respiratory symptoms among asthmatic children were associated with PM_2.5_ concentrations (OR = 1.08 [95% CI: 1.03, 1.14] for a 10-µg/m^3^ increase in PM_2.5_ on the same day). However, in their study, no significant association was observed between wheezing and exposure to PM.

Previous studies that found the significant effects of PM on respiratory health had been conducted in areas with high concentrations of air pollutants.^8^^,^^31^ Some studies observed no significant associations between PM concentration and respiratory symptoms in areas with low levels of air pollution.^5^^,^^7^^,^^12^ Romieu et al^32^ reported that the effects of PM on changes in PEF in asthmatic children were not significant in an area with low ambient levels of PM. In our study, the concentrations of stationary-site PM_2.5_ were not associated with changes in PEF in the asthmatic children, although they were weakly associated with wheezing. This study was conducted in a suburban city without major sources of air pollution, and the concentration of stationary-site PM_2.5_ during the study period was considerably lower (average PM_2.5_ concentration = 19.1 µg/m^3^) than the PM_2.5_ levels in areas where the significant effects of PM_2.5_ were previously found. This may explain why we were unable to detect its effects on the changes in PEF.

The concentration of PM_2.5(LD)_ at the entrance of the hospital was significantly associated with changes in PEF and wheezing, while the concentration of PM_2.5(LD)_ in the hospital was more consistently and strongly associated with these symptoms. The maximum decreases in PEF in relation to a 10-µg/m^3^ increase in the 24-h concentration of indoor PM_2.5(LD)_ were -2.86 L/min in the morning and -3.59 L/min in the evening. These changes in PEF in relation to an increase in PM_2.5(LD)_ concentration were greater than the changes observed in previous studies.^9^^,^^15^ The prevalence of wheezing was also significantly associated with indoor PM_2.5(LD)_ concentration, although the observed ORs for wheezing were considerably small (ORs in relation to a 10-µg/m^3^ increase in the 24-h concentration of indoor PM_2.5(LD)_ were 1.014 and 1.025 in the morning and evening, respectively). The concentrations of indoor and outdoor PM_2.5(LD)_ varied considerably during the study period. The analyses using quartiles of each PM showed that the prevalence of wheezing increased in relation to exposure to high concentrations of indoor PM_2.5(LD)_ or stationary-site PM_2.5_.

It is difficult to compare the results of our study with those of other studies because of the differences with regard to various factors, such as race, age, severity of asthma, and concentrations of co-pollutants, which can influence PEF. In most of the previous studies, the data on air pollutants was collected at central regional sites, and all the subjects were usually assigned to uniform exposure.^6^^,^^12^^,^^14^ Thus, exposure misclassification is expected to diminish the accuracy of exposure-response estimates, possibly leading to a null effect. We measured the concentrations of PM inside and outside the hospital in which the subjects stayed for a long period of time. Because the concentration of indoor PM was estimated from measurements taken at 3 sites in the hospital, it is conceivable that personal exposure to PM in the children has been evaluated much more accurately in the present study than in previous studies. In addition, daily measurements of PEF were conducted regularly using a spirometer, under the guidance of trained nurses, and wheezing was assessed based on auscultation. Therefore, we believe that our results reflect the actual exposure-response relationship.

In our study, the decreases in PEF and increases in wheezing in relation to increases in PM_2.5(LD)_ concentration were more remarkable in the evening than in the morning. Roemer et al^14^ found that decreases in PEF in relation to increases in PM concentration were larger in the evening than in the morning. However, other studies have reported that decreases in PEF associated with exposure to PM were greater in the morning than in the evening.^31^^,^^33^ Thus, there have been no consistent findings on the difference in the effects of exposure to PM between morning and evening. PEF values in the evening appear to be affected by daily activities during the daytime. In this study, all the subjects were children who were hospitalized, and their habits were almost identical. We found that the effects of confounding factors other than air pollution were small, thereby allowing detection of the marked effects of indoor PM_2.5(LD)_ concentration on PEF and wheezing.

To assess the temporality from exposure to PM to the changes in PEF and the occurrence of wheezing, the lag structure of the associations has been examined in many reports.^5^^,^^8^^,^^12^^,^^33^^,^^34^ Decreases in PEF have been reported to be more relevant to the concentration of PM after a 1-d lag than that on the same day.^8^ In a panel of asthmatic children in another study, a significant relationship between lower respiratory symptoms and the 5-d mean concentration of PM_10_ was found, but the associations for 0-2 d lags were not significant.^5^ Desqueyroux et al^34^ reported that asthma attacks in adults with severe asthma were associated with PM_10_ concentrations for 3-5 d lags, but such association for a 1- or 2-d lag was not significant.

In the present study, significant decreases in PEF were observed in relation to outdoor PM_2.5(LD)_ concentration for 1- and 2-d lags in the morning and 0- and 1-d lags in the evening. Wheezing in the morning was related to outdoor PM_2.5(LD)_ concentration only for 2-d lags. These findings are consistent with the results of previous reports,^5^^,^^8^^,^^34^ which show that the effects of PM differ in relation to the number of lag days. The concentrations of indoor PM_2.5(LD)_ for 0-3 d lags were consistently associated with both PEF and wheezing, and such associations became gradually weaker as the number of lag days increased. Thus, lag periods from exposure to PM to observed effects were different for indoor and outdoor PM concentrations. These results suggest that indoor PM may affect asthmatic children more easily than outdoor PM. However, the concentrations of stationary-site PM_2.5_ were not significantly related to PEF for upto 3-d lags.

Some researchers have shown that concentrations of indoor PM often differ from those found in outdoor air.^25^^,^^26^ Long et al^24^ assessed the *in vitro* toxicity of indoor and outdoor PM_2.5_ collected in Boston-area homes, and suggested that indoor-generated particles may be more bioactive than outdoor particles. The differences may be driven by the types of materials used for building.^35^ It was also reported that the concentrations of indoor PM were similar to the outdoor levels when air change was conducted frequently. However, indoor sources might seriously affect the concentrations of indoor PM.^36^ In the present study, the mean concentration of indoor PM_2.5(LD)_ was higher than that of outdoor PM_2.5(LD)_. The concentration of indoor PM_2.5(LD)_ did not correlate with the concentration of stationary-site PM_2.5_, although it showed a weak correlation with outdoor PM_2.5(LD)_ concentration. Moreover, indoor PM_2.5(LD)_ reached a higher concentration during the nighttime than during daytime. These results suggest that the sources of indoor PM differ from those of outdoor PM. However, in the hospital, no typical sources of PM, such as smoking and cooking, were present, and we could not identify the major sources of indoor PM. In addition, the factors that account for high concentrations of indoor PM_2.5(LD)_ during the nighttime remain unknown. Some allergens, such as house dust mites, might contaminate the indoor environment.^37^ The origin and characteristics of PM in the hospital should be further evaluated.

With respect to the lag structure for every 12 h before PEF measurement, indoor PM_2.5(LD)_ concentrations during both the daytime and nighttime were significantly associated with PEF in children. Compared to the nighttime concentrations, the concentrations of indoor PM_2.5(LD)_ during the daytime were more strongly associated with changes in PEF. Similar results were observed with regard to the effects on wheezing. This may reflect the difference in the concentrations of indoor PM_2.5(LD)_ between daytime and nighttime. Alternatively, the effects of nighttime PM concentration on children might be lesser than those of daytime PM concentration because they were asleep for most of the time during the night.

Several studies have used size-fractionated PM data to compare the effects of fine and coarse fraction particles. Schwartz et al^16^ reported that PM_2.5_ may have more adverse effects on respiratory symptoms and pulmonary functions among schoolchildren than PM_2.5-10_. A study on Chinese schoolchildren reported that during the winter heating season, the effects of fine particles on pulmonary functions were greater than those of coarse particles.^38^ With respect to the effects on PEF in asthmatics, Romieu et al^9^ and Pekkanen et al^13^ reported comparable results for PM_2.5_ and PM_10_, while Peters et al^15^ found slightly greater effects for PM_2.5_. In Japan, particulate air pollution is usually assessed based on the concentration of suspended particulate matter (SPM), which is the fraction of particles with diameters less than 10 µm. However, the method of measuring SPM is different from that of PM_10_ measurement in foreign countries, and the concentration of SPM cannot be regarded as that of coarse particles. Therefore, we did not consider the effects of coarse particles. An additional study is necessary to evaluate the effects of coarse particles in Japan.

Several previous studies have reported the relationships between air pollutants and medication use in asthmatic children.^4^^-^^7^^,^^39^^,^^40^ In a panel of children, most of whom had asthma, Gielen et al^5^ reported an association between PM concentration and medication use. All the subjects in our study had severe asthma and used maintenance medication, including ICS, daily. Therefore, in this study, we did not examine the associations with medication. The medication for asthma might obscure the effects of exposure to PM, although the measurements of PEF and the assessments of wheezing were conducted immediately before medication. Delfino et al^11^ reported that associations between asthma symptoms and exposure to PM were significant in only the group of children who were not under anti-inflammatory medications. However, Gent et al^41^ reported that children using maintenance medication were particularly vulnerable to ozone. In our study, the concentrations of indoor PM_2.5(LD)_ were significantly associated with PEF and wheezing. These findings are compatible with the results by Gent et al.^40^

In conclusion, among children from this panel study, we found no obvious association between the concentrations of stationary-site PM_2.5_ and PEF or wheezing. However, even at low levels of ambient air pollution, the concentrations of indoor and outdoor PM_2.5(LD)_ were associated with PEF and wheezing among asthmatic children. The consistent and strong associations of PEF and wheezing with indoor PM_2.5(LD)_ concentrations suggest that it is desirable to estimate exposure to PM in the environment where the subjects spend most of their time.
